# Network Pharmacological Study of *Achyranthis bidentatae* Radix Effect on Bone Trauma

**DOI:** 10.1155/2021/5692039

**Published:** 2021-03-06

**Authors:** Liying Wu, Youguo Hao, Chuanqiang Dai, Zhibang Zhang, Munazza Ijaz, Sobhy M. Ibrahim, Ghulam Murtaza, Zhiguang Yao

**Affiliations:** ^1^Department of Orthopedics, Fushun Mining Bureau General Hospital of Liaoning Health Industry Group (The Seventh Affiliated Hospital of China Medical University), Fushun, Liaoning 113008, China; ^2^Department of Rehabilitation, Shanghai Putuo People's Hospital, Shanghai 200060, China; ^3^Department of Orthopedics, The First People's Hospital of Ziyang, Ziyang, Sichuan 641300, China; ^4^Department of Bone and Joint Surgery, Qinghai Fifth People's Hospital, Xining, Qinghai 810007, China; ^5^Institute of Molecular Biology and Biotechnology, The University of Lahore, Lahore, Pakistan; ^6^Department of Biochemistry, College of Science, King Saud University, P.O. Box: 2455, Riyadh 11451, Saudi Arabia; ^7^Department of Pharmacy, COMSATS University Islamabad, Lahore Campus, Lahore 54000, Pakistan; ^8^Graduate School of Clinical Medicine, Zhejiang Chinese Medical University, Hangzhou, Zhejiang 310053, China; ^9^Department of Orthopedics, Haining Hospital of Traditional Chinese Medicine, Haining, Zhejiang 314400, China

## Abstract

**Purpose:**

Bone trauma is a clinical condition that afflicts the majority of the world's population. For the management of bone trauma, the underlying mechanisms of the drugs effective for bone healing are deemed necessary. *Achyranthis bidentatae* Radix (ABR) is a popular alternative medicine recommended in the treatment of bone trauma and injury, yet its mechanism of action persists to be vague. This study was conducted for the evaluation of the mode of action of ABR through network pharmacology in treating bone trauma.

**Methods:**

An extensive survey of published works led to the development of a drug-target database, after which multiple protein targets for bone trauma were discerned. The protein-protein interaction network was developed by utilizing the STITCH database and gene ontology (GO) enrichment analysis using Cytoscape and ClueGO. Moreover, docking studies were performed for revealing the affinity of various ingredients with IL6.

**Results:**

The extensive literature survey yielded the presence of 176 components in ABR, and 151 potential targets were acquired. Scrutinization of these targets revealed that 21 potential targets were found to be associated with bone trauma. Out of which, some remarkable targets such as IL6, MAPK14, MAPK8, SRC, PTGS2, and MMP2 were observed to be associated in the functional interaction of ABR. According to docking results, several ingredients of ABR such as Baicalien, Copistine, Epiberberine, Kaempferol, and Palmatine have the lowest docking scores (range between -6 and -7).

**Conclusions:**

The results of the study elucidated that ABR can positively be utilized for the management of bone trauma, which can be mediated by multiple molecular mechanisms such as ERBB2 signaling pathway, positive regulation of oxidoreductase activity, JNK cascade pathway, multicellular organism metabolic process, T cell costimulation, and the positive regulation of MAPK activity. The findings also suggest that several ingredients of ABR such as Baicalien, Copistine, Epiberberine, Kaempferol, and Palmatine have good affinity with IL6, suggesting the promising potential of ABR in treating bone trauma, likely through IL6.

## 1. Introduction

*Achyranthis bidentatae* Radix (ABR) is a traditional herb that is often used to treat bone trauma and bone injury [1]. Recent research has established Traditional Chinese Medicines (TCM) therapy and its use with the reduced probability of bone injury, such as fracture and osteoporosis in various studies [2]. ABR induces osteogenesis of mesenchymal stem cells (MSCs) of the bone marrow via activating the extracellular signal-regulated kinase (ERK) signaling cascade and inhibiting nuclear factor kappa B (NF-*κ*B) pathway in rats [3]. However, its association with different pathways was elucidated concerning single compounds, and the multiple drug-target systems remained vague. In the human body, the bone is reported to be a remarkable organ regulated by its dynamic remodelling through its entirety, governed by the action of various cells involved in these stages [4]. Osteoblasts are those cells which have been differentiated from MSCs through the action of transcription factors like Runt-related transcription factor 2 (Runx2) and Osterix (Osx) and are secreted into the bone matrix which ultimately leads to the induction of bone mineralization [4]. In the eventual stage of the remodelling process, they either undergo programmed cell death or tend to be consolidated into the bone matrix during calcification [5].

Bone functions are accomplished via two coupled processes, i.e., osteoclast-mediated bone resorption, followed by osteoblast-mediated bone formation. Both processes occur homeostatically in adults. The imbalance of this homeostasis leads to bone disorders [6]. The differentiation of osteoclasts is mediated by various factors such as Macrophage Colony Stimulating Factor (M-CSF) and receptor activator of NF-kappa B ligand (RANKL), where the cells initiate fusion and development into mature and active osteoclasts through the action of the latter [7]. Loss of bone and its resultant repair is deemed to be significant issues in bone-related studies [8]. To comprehend the bone loss and repair mechanism of traumatic injuries, it is crucial to understand the mechanism that is carried out for the whole process [8]. Bone trauma, injury, and bone loss pertains to an issue of great economic as well as clinical significance, as more than 100, 000 cases of bone fracture are reported every year in the United States [8]. Bone healing is an amalgamation of many stages that are directly and indirectly involved in the mediation of various pathways that elicit the recovery process [9]. Almost immediately after bone trauma is induced, a hematoma is produced which comprises majorly of bone marrow MSCs, while also initiating an inflammatory response which sequesters immunomodulatory molecules at the site of the injury [9]. The initial onset of proinflammatory molecules like tumor necrosis factor-*α* (TNF-*α*) and interleukins (ILs) has shown to encourage angiogenesis and mediate necessary inflammatory molecules [9]. Furthermore, TNF-*α* is reported to induce osteogenesis of MSCs *in vitro* and regulated through the activation of receptor molecules such as TNF receptor 1 and TNF receptor 2, both of whom are actively found on both osteoclasts and osteoblasts, respectively [9]. Remarkably, the former is reported to be expressed in the bone while the latter only specifically expresses itself after bone injury, which is indicative of its role in bone regeneration [10]. After the advent of modern sciences and technology, network pharmacology has reportedly been hailed as the trendsetting outlook to the disease-gene-target-drug interaction [9]. It works by the systematic and comprehensive characterization of the mechanism and the connection of drugs, thus revealing their therapeutic effect on the human body [11]. The evolving techniques used in bioinformatics offer the opportunity to openly determine and evaluate the mechanism of a wide variety of TCM-based traditional drugs in treating a myriad of diseases [11]. Proteins in the interaction network are involved in signaling and serve a key role in maintaining the regulation of various biological functions [12].

The present study is aimed at analyzing the therapeutic mode of action of ABR through the aid of network pharmacology. As of now, sparse knowledge and information exist on the mechanism of treatment of bone trauma through ABR, which calls for the deeper evaluation of the protein targets and their interactions. In this study, the drug-target interaction was studied by the construction of a protein-protein interaction network. Moreover, Gene Ontology (GO) enrichment analysis was used to explore biological functions associated with the mode of action of ABR [13]. Therefore, this study could prove essential for the experimental study that may examine the effect of ABR on bone trauma.

## 2. Methodology

**Figure 1:**
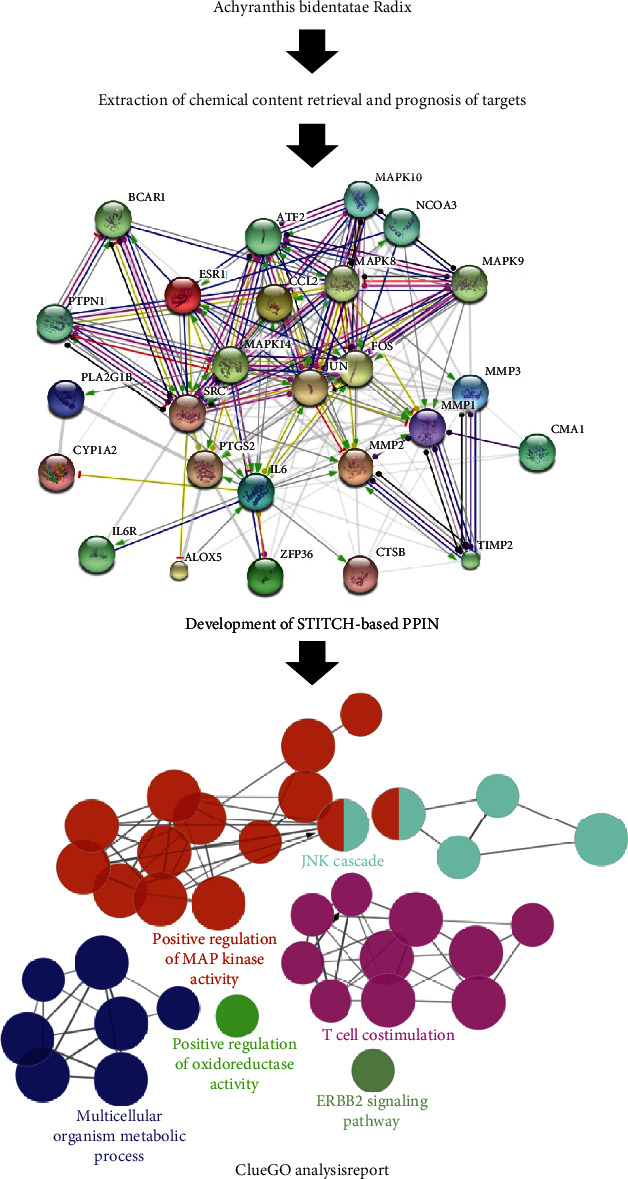
The demonstration of the systematic process used in this study.

For the network development and its subsequent analyses, STITCH database was employed to elucidate the effect of ABR on bone trauma.  Figure 1 denotes the retrieval of the chemical compounds of ABR, and the potential protein targets in *Homo sapiens* in step 1, followed by the development of a protein-protein interaction network (PPIN) and GO analysis in step 2. Lastly, the GO term analysis was conducted by using Cytoscape software and its plug-in ClueGO to elucidate the mechanism of action of ABR against bone trauma at a molecular level.

### 2.1. Extraction of Chemical Contents and Their Targets

ABR formula was searched according to the literature reported upon it, including the Traditional Chinese Medicine Systems Pharmacology Database and Analysis Platform (TCMSP) (https://tcmspw.com/tcmsp.php) [14]. TCMSP database was developed based on the knowledge of system pharmacology. It comprises about five hundred herbs, thirty thousand compounds, three thousand protein targets, and eight hundred relevant diseases. This database not only provides information about different pharmacokinetic features of compounds such as drug likeliness and oral bioavailability but also develops networks such as a compound-target network. It has helped the researchers to accelerate the process of drug discovery and development by integrating herbal medicines with modern medicines [14]. The chemical components were examined and the protein targets were acquired from reported literature, including review and research articles, as well as TCMSP database, after which the genes associated with ABR and its biological functions were assessed from UniProtKB (https://www.uniprot.org) and KEGG (https://www.kegg.jp/), and the molecular targets were examined for the association to bone trauma.

### 2.2. Network Construction and Analyses

For the investigation of ABR and its therapeutic mechanism of action, STITCH 5.0 database (http://stitch.embl.de/) [15] was used, which is an online hub of reported protein structures and their resultant interactions which may be mediated directly or indirectly. The subsequent information of these interactive networks is acquired from four sources which comprise gene studies, coexpression, high-throughput experimental data results, and text mining. Currently, this database holds information of more than 9.5 million proteins, isolated from more than 2500 organisms. This interaction between chemical components of a particular drug and their associated targets was then evaluated by the construction of an interactive network to evaluate the mode of action of ABR, and its pharmacological constituents. Protein-protein interaction network was established by adding TCMSP-origin protein targets to STITCH database, by selecting the options of “Multiple Items by Names/Identifiers” and “*Homo sapiens*.” Minimum required interaction score and a maximum number of interactors to show in the first shell were set at 0.004 and 10, respectively. Action view of the acquitted network was saved for further use. Furthermore, various statistical and biological aspects of the network were also downloaded. Additionally, SymMap database (https://www.symmap.org) [16] was used for the construction of a network between ABR, its active components, and its associated targets, as well as the disease itself.

### 2.3. GO and Pathway Enrichment Analyses

In the next phase of the study, the characteristic biological functions of the chemical components were analyzed and studied using GO enrichment analysis for the identification of target genes in a structural fashion, categorized according to the biological terms. This analysis was deemed useful for the investigation of the mode of action of ABR against bone trauma. Cytoscape (version 3.4.0) and its plugin-ClueGO [17], with a level of significance set for 0.05, were also utilized for the elucidation of a protein and its associated target network for the better understanding of the biological pathways of ABR. Kappa score for pathway network connectivity was set at 0.4 (a cut-off point). Furthermore, a two-sided test with Bonferroni correction was used, with a medium network which reveals GO terms with GO levels (GO tree interval) in the range of 4-8. Lastly, the visualization of the functional interaction and network was performed by using an organic layout algorithm from Cytoscape.

### 2.4. Docking Study

The docking of ten compounds (Rutin, Aregenal, Astragalin, Baicalien, Copistine, Epiberberine, Hyperin, Kaempferol, Myristic acid, and Palmatine) with one of the targets (interleukin-6) was carried to computationally search an appropriate ligand that fits the binding site of protein-based on its geometry and energy [18].

For ligand preparation, all the ligand (compound) structures were searched in PUBCHEM and saved in MDL Molfile v3000 format. The file was then opened in AutoDock-4.2, nonpolar hydrogen atoms were merged, torsions value was set to default while Kollman charges, and Gasteiger charges were added using default parameters.

To find the role of interleukin-6 in osteoporosis, the protein structure of IL6 was searched and downloaded from Protein Drug Data Bank (http://www.rcsb.org). The 3D crystal-structure IL6 with protein data bank id 1ALU is downloaded in PDB format. The 3D structure of the protein was then opened in Autodock-4.2, water molecule was removed, polar-hydrogen atoms were added, and Kollman charges were assigned to both the proteins.

Docking evaluation of IL6 with all the compounds was performed through Auto-dock 4.2. All the compounds were separately docked with IL6. Before running docking on both the ligand and receptors, Grid-box was set for blind docking and saved.

Various grid parameters were assigned to ensure the ligand binding to the active site of protein through navigation, and the output was saved in the format of grid parameter file (GPF). The AutoGrid execution was accomplished by using the AutoGrid and GPF files as input, transforming to the grid log file (GLG), and launching of grid.

For docking, a genetic algorithm was selected as searched parameter while setting the number of runs to 30, and for output, Lamarckian-GA4.2 was selected, and the output was saved in docking parameter file (DPF) format. The rest of the docking parameters was set to default. The AutoDock execution was accomplished by using the AutoDockable and DPF files as input, transforming to the docking log file (DLG) format, launching of docking, followed by analysis of the results. Binding energy was the basic criteria for the ranking of results. The PDBQT format was used for saving the findings. The lowest binding energy complex of ligand and protein was saved in PDB format and used for further analysis.

Ligand-protein interaction was further studied by using Discovery Studio 2020 from BIOVIA.

## 3. Results

### 3.1. Extraction of Chemical Components and Their Associative Targets

The cumulative number of compounds acquired from ABR was 176 (Supplementary Table 1). An extensive literature survey revealed 151 potential protein targets for these chemical compounds. Preliminary experimental findings [3, 19–22] stated that ABR could be potentially employed for the effective treatment of Central Nervous System (CNS) disorders and pain. Consequently, 21 of 151 targets of ABR were reported to possess pharmacological activity against these disorders. The 21 screened targets (Supplementary Table 2) were assimilated using UniProt database-mapping (https://www.uniprot.org/). The acquired protein targets were assessed for their activity, of which 16 out of 21 targets were reported to be associated with humans.

### 3.2. Development of Interaction Network and Its Analysis

**Figure 2:**
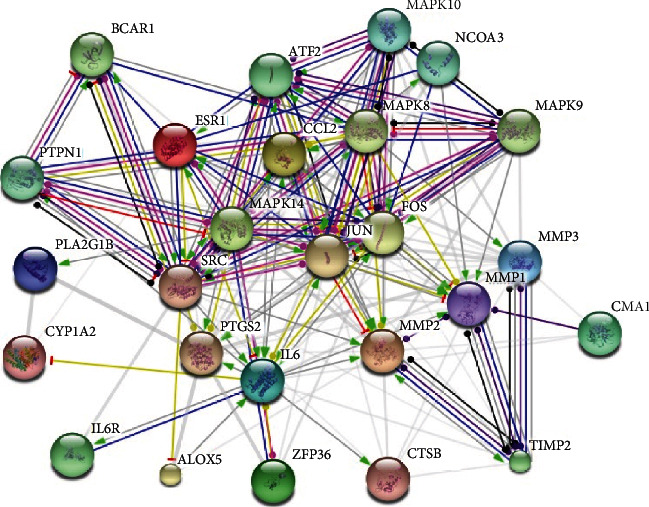
Action view of the PPIN network of ABR targets. Action type is denoted by colored edges: activation, inhibition, binding, catalysis, phenotype, posttranslational modification, reaction, and transcriptional regulation. Action effects are shown by the following: positive, negative and unspecified. Note: ESR1: estrogen receptor 1; MMP2: matrix metallopeptidase 2; CCL2: chemokine (C-C motif) ligand 2; MAPK14: mitogen-activated protein kinase 14; ZFP36: zinc finger protein 36, C3H type, homolog (mouse); CMA1: chymase 1, mast cell; IL6: interleukin 6 (interferon, beta 2); MMP3: matrix metallopeptidase 3 (stromelysin 1, progelatinase); PLA2G1B: phospholipase A2, group IB (pancreas); MMP1: matrix metallopeptidase 1 (interstitial collagenase); CYP1A2: cytochrome P450, family 1, subfamily A, polypeptide 2; CTSB: cathepsin B; SRC: v-src sarcoma (Schmidt-Ruppin A-2) viral oncogene homolog (avian); PTGS2: prostaglandin-endoperoxide synthase 2 (prostaglandin G/H synthase and cyclooxygenase); JUN: jun protooncogene; ALOX5: arachidonate 5-lipoxygenase; FOS: FBJ murine osteosarcoma viral oncogene homolog; MAPK8: mitogen-activated protein kinase 8; MAPK9: mitogen-activated protein kinase 9; BCAR1: breast cancer antiestrogen resistance 1; TIMP2: TIMP metallopeptidase inhibitor 2; IL6R: interleukin 6 receptor; ATF2: activating transcription factor 2; PTPN1: protein tyrosine phosphatase, nonreceptor type 1; NCOA3: nuclear receptor coactivator 3; MAPK10: mitogen-activated protein kinase 10.

**Figure 3:**
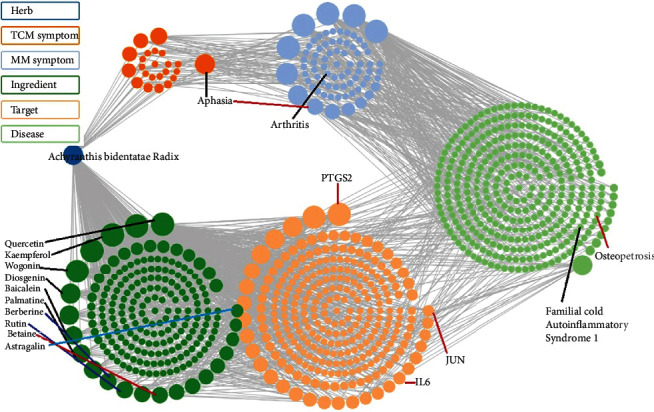
SymMap retrieved network of herb-compound-diseases-targets, showing clear evidence of ABR association with bone trauma and the involved ingredients and protein targets.

STITCH database was employed for the construction of a PPIN (Figure 2), which comprised of 26 standardized target proteins. The probability value was set at 0.400, i.e., a medium value. The network demonstrated 26 nodes and 146 edges. From these obtained 26 nodes, 10 were attributable to functional interactions. Nodes of a network tend to denote the specific protein targets or the related genes that are working in association with the chemical compounds. The interlinkage of different genes and their pairs are denoted by edges (lines) in the network. According to the obtained values of PPIN statistics, the PPIN enrichment *p* value was negligibly small, i.e., 0.001. The approximate number of edges were observed to be 38 in the case where the nodes were chosen randomly. If a small PPIN enrichment value is achieved in the network, this shows that the nodes and edges are not random and are significant, respectively. The mean node degree and the clustering coefficient in a PPIN denote the average interactions of a respective target at a threshold value and the degree of connectedness of the PPIN nodes, respectively. In this PPIN network, the average node degree and its clustering coefficient values were observed to be 11.2 and 0.742, respectively. The connectivity of the network is proportionate to the high value of the clustering coefficient and vice versa. The relative value of interactions of a particular node in a PPIN is denoted as the node degree value, characterized by a quantifiable attribute of a node. Hubs are those nodes which are marked by elevated interactive values than that of the mean node degree. Eleven hubs of this PPIN were found to be associated with functional interactions as their node degree is greater than the mean node degree of the network, i.e., 11.2. Amongst these proteins, IL6 and SRC demonstrated the greatest node degree, i.e., 21, after which JUN and FOS showed node degrees of 20 and 18, respectively. Furthermore, other proteins such as MMP2, MAPK14, PTGS2, and MAPK8 all demonstrated the node degree value of 17. Each node and its degree value are elucidated in Table 1. These hubs and their functional interactions with other nodes have been reported in various kinds of literature regarding bone trauma.  Table 2 describes the functional nodes which are active in this PPIN, which demonstrates that ABR interaction can result in the activation and binding of all stated functional proteins, whereas FOS, TIMP2, ATF2, and PTPN1 could be inhibited by the action of ABR. Moreover, ABR is involved in the catalysis process of almost all functional proteins except for IL6R, and the posttranslational modification processes of all these except for TIMP2, IL6R, and NCOA3, respectively, as observed in Table 2.  Table 3 illustrates various functional enrichments in the PPIN. Furthermore, Figure 3 presents the herb-compound-diseases-targets network.

### 3.3. GO and Pathway Enrichment Analyses

**Figure 4:**
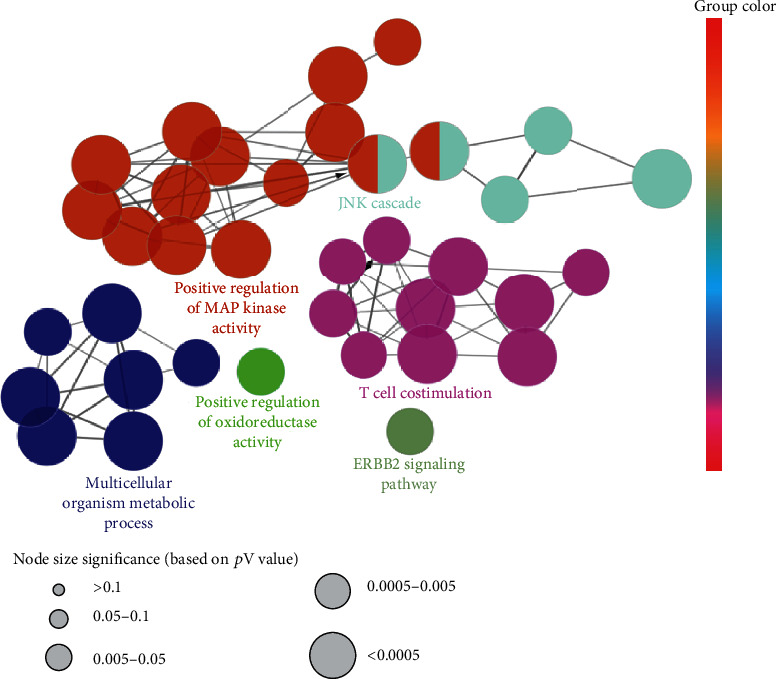
Networks consolidated on a functional basis obtained for potential targets of ABR through ClueGo. Each functional group is denoted by the annotation of the most important term. Groups that are interacting on a functional basis may converge with each other.

**Figure 5:**
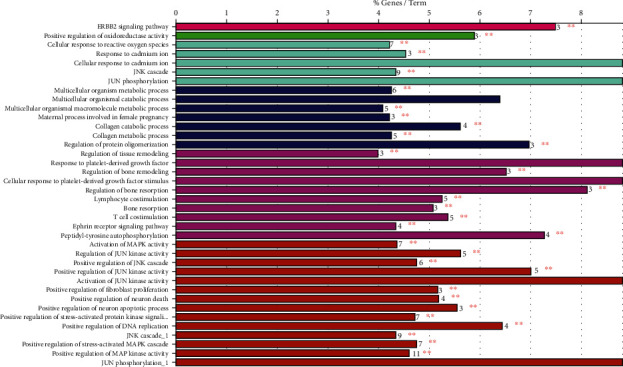
Multiple GO terms obtained via GO analysis.

Targets of ABR were evaluated by using Clue-GO enrichment analysis. GO terms were used for the characterization of biological compounds and pathways. The subsequent analysis led to the elucidation of 176 GO terms, which were then divided into 39 subgroups, primarily involved in the ERBB2 signaling pathway, positive regulation of oxidoreductase activity, JNK signaling cascade, multicellular organism metabolic process, T cell costimulation, and positive regulation of MAPK activity (Table 4, Figures 4 and 5). These study findings prove to be significant in explaining the mechanism of ABR.

### 3.4. Docking Study

The docking findings are presented in Table 5, which reveals that Baicalien, Copistine, Epiberberine, Kaempferol, and Palmatine have the lowest docking scores, i.e., in a range of -6 to -7. It indicates their good affinity with interleukin-6. The number of hydrogen bonds of these five compounds with interleukin-6 was 6, 6, 2, 6, and 3, respectively (as shown in Table 5).

## 4. Discussion

*Achyranthis bidentatae* Radix (ABR), an herb used customarily in alternate medicine, is administered when treating bone diseases and bone trauma [3]. However, the mechanisms through which it is rendered effective remain elusive. For the elucidation of the mode of action of ABR against bone trauma and its healing effect, an *in silico* study based on network pharmacology was designed. Network pharmacology employs the use of pathways which deduces the association of protein compounds and the genes affiliated with the disease, thus explaining in intricate detail the complexities that arise in various organisms, drugs, along with many diseases from a network standpoint. This ring is similar to the holistic approach employed by TCM, which affirms the harmonious use of TCM with modern methods of systems biology [23]. In this study, multiple approaches were used such as the retrieval of various targets for drugs, the development of interactive networks, and the analyses of diverse pathways. A cumulative 151 protein targets of ABR had been acquired consequently of the extensive search. As a result, GO analyses were performed which subsequently affirmed the activity of ABR against bone injury and trauma. Moreover, pathway enrichment analysis revealed the role of ABR in the regulation of various pathways which act congruently with multiple therapeutic modules.

Multiple compounds like copsitine, berberine, myristic acid, wogonin, baicalein, baicalin, (R)-Allantoin, caprylic acid, rutin, kaempferol, betaine, hyperin, astragalin, palmatine, and quercetin have been elucidated to be the major bioactive compounds in ABR (Table 6), as they tend to exhibit their protective effects against bone trauma and pain, based on various *in vitro* and *in vivo* studies. Berberine is an alkaloid which exerts a protective effect against the induced bone destruction via inhibiting TNFRAF-6 and Ca2+-calcineurin nuclear factor of activated T cell 1 (NFATc1). This protective effect leads to the inhibition of osteoclastogenesis, thereby inhibiting bone destruction [24]. Xie et al. [25] reported the positive effect of berberine in diabetic osteoporosis, which was achieved through the antioxidant action of berberine and the downregulation of DNA damage in HFD-induced diabetic rats. Baicalin is reported to be compound with a crucial role in the treatment of cancer-induced bone pain, via the upregulation of transient receptor potential vanilloid 1 (TRPV1), thus preventing the progression of bone pain in cancer [26]. The mechanism of baicalein in bone pain in cancer was studied by Hu et al. [27], who reported that the compound might be effective by inhibiting P-P38 and p-JNK MAP kinase signaling pathway, while the protective analgesic effect may be through the inhibition of various cytokines like IL6. Kim et al. [28] also reported the inhibition ability of osteoclasts by inducing the inhibition of RANKL-induced activation of various signaling precursors. Osteoarthritis caused by trauma was also observed to be alleviated with the administration of myristic acids in rats [29]. Rutin was reported for the protective effect it exerts in the case of ovariectomy-induced loss of trabecular bone in rats, by decreasing the process of bone resorption and regulating an increase in osteoblastic activity [30]. The combinative effect of quercetin and rutin was observed to be positive for bone health, due to the marked elevation in markers of bone development and genesis and the subsequent decline in bone resorption markers [30]. Kaempferol isolated from *Kaempferia galanga* L. elucidated the protective and positive action of kaempferol leading to elevated bone resorption and genetic expression of osteogenesis [31]. Furthermore, Nepal et al. [32] reported that kaempferol inhibited the phosphorylation of P38, ERK½, CFOS, and JNK-MAPK and subsequently inhibited the generation of osteoclasts. Kaempferol was also effective in decreasing the interleukin 1*β*-stimulated proinflammatory cytokines, indicating that it had a remarkable effect against arthritis and osteoporosis, stimulating bone health [33]. Astragalin was reported to inhibit the release of proinflammatory cytokines and the expression of MMPs in induced arthritis in mice [34].

In the protein-protein interaction network, the main hubs which were reported were IL6, SRC, JUN, FOS, MMP2, MAPK8, MAPK14, and PTGS2, which are already reported to be actively involved in the treatment of bone trauma and injury. SRC is greatly expressed in the membrane of osteoclasts, as well as in the cells of various organelles, suggesting its intricate involvement in various cellular functions. In osteoclasts, SRC and its subsequent signaling pathway tend to get activated after the binding of cells in the bone matrix, where it interacts with various molecules through receptors that mediate signaling pathways [35]. IL6 serves a key role in the modulation of mediatory functions in various stages of bone repair [36]. In bone trauma, IL6 reigns over the action and activity of various immune cells and promotes angiogenesis [37]. In various repair stages, IL6 mediates the intramembrane and endochondral ossification and initiates the modelling of fracture wounds, thus affirming that IL6 transsignalling greatly improved healing in the bone after severe bone trauma [38]. IL6 also induces changes in bone remodeling by the induction of osteoblasts and JAK/STAT3 pathways, which is also reported to be activated by the action of C-SRC in immature osteoblasts [39]. These findings support our results of docking study. The vitality of JNK (c-Jun N-terminal kinase) was elucidated in rheumatoid arthritis, where it was observed that it is a significant precursor in MAPK pathway [40]. Jun performs an active part in the acceleration of bone growth and healing after severe trauma, by mediating the osteoprogenitor cells and regulating the AP-1 transcription factor, an immunomodulatory factor both common in JUN and FOS families [41, 42]. MAPKs are the enzymes that are implied in regulating extracellular stimulus factors which are transduced into various processes of the cell. In many instances, they play their role as a signaling hub where multiple and a diverse range of pathways overlap to activate a peculiar MAPK required according to the situation. MAPK14, also known as p38 MAPK, is very important for osteoblasts for maintaining their reported function and activity in adult bone and surrounding tissues [43]. Various studies have demonstrated that p38 deletion inhibits osteoblast terminal differentiation and ultimately affects bone composition and regulation. In vitro studies have shown that p38 integrates various external stimuli, including signaling precursors like cytokines, and the BMP/TGF*β* pathways [44].

The analysis of the pathway enrichment demonstrates the association of almost all GO terms with bone trauma. Moreover, three GO terms, namely, ERBB2 signaling pathway (GO ID: 38128) [45], JNK cascade (GO ID: 0007254) [43, 44], and positive regulation of MAP kinase activity (GO ID: 43406) [46], are reported to be significantly associated with bone trauma and injury. The ERBB2 signaling pathway is a network of molecular signals that are initiated by binding of a ligand to ERBB family member of cell surface receptors, where the signal is transduced by ERBB2 [45]. The pathway terminates with the regulation of downstream processes in the cell. Its receptors are not able to bind to ligands but tend to act as signal-augmenting tyrosine kinase. Positive regulation of MAP kinase activity denotes any process that activates or increases the frequency or rate of MAP kinase activity [43, 44]. The JNK cascade refers to the protein kinase cascade that comprises JNK, JNKK, and JUN3K kinases that altogether transmit a signal within a cell. The mechanism of bone healing in trauma and injury is a complex and intricate process that obeys a multitude of specific regenerative paths and involves the expression of a diverse range of genes [46]. The MAPK pathway is reported to have an established role in skeletal development, which has been demonstrated by in vivo studies. The p38 MAPK pathway is important for the differentiation of osteoblasts, where it acts as a downregulated signal which is activated by TGF-*β* and BMP responsive kinase, TAK1. In the case of TAK1 deficiency, the p38 intermediates are deemed to be attributable to it which results in decreased bone mass [47]. In our study, the positive regulation of MAP kinase activity was found to be associated with many genes including CSK, KRAS, MAP2K4, MAP2K7, MAP3K11, MAPK10, PLA2G1B, PTPN1, SHC1, SRC, and TAOK3. Similarly, the JSK signaling cascade is associated with MAP2K4, MAP2K7, MAP3K11, MAPK10, MAPK8, MAPK9, PTPN1, RB1CC1, and TAOK3. It is well established from previous studies that MAPK signaling pathway is indeed one of the most vital signal transduction systems comprising of three subpathways, namely, ERK, JNK, and p38. Amongst the three, the ERK pathway has been touted as a stimulant for the expression and regulation of growth factors like VEGF, leading to osteogenic differentiation. A study shed light on the possible mechanism of the ERK pathway, which described the signaling pathway to be activated by HMGB1, for the induction and migration of fibroblasts to accentuate wound healing [48, 49]. The reported literature has elucidated the function of ABR to be effective in mitigating bone trauma and injury through modulating different immune responses that treat and reduce damage to the bone tissue. The findings of our study are elemental in deducing the mode of action of ABR in the treatment of bone trauma as well as wound or bone healing. However, they are presented in a preclinical fashion that calls for clinical trials of ABR against bone trauma, to correlate the results with evidence-based medicinal approaches.

## 5. Conclusions

For the establishment of an intricate evaluation of potentially active drugs and their target proteins, various aspects of an *in silico* study are bound together by pharmacokinetics and pharmacodynamic features for the subsequent network pharmacology analyses. The elucidation of different mechanisms of action of traditional drugs and their interactions with various biological systems is analyzed, which can help in understanding the therapeutic features of various drugs. In our work, network pharmacology analysis was conducted to understand the mode of action of alternative medicine, *Achyranthis bidentatae* Radix (ABR). The study design consisted of the search for potential chemical targets, their subsequent associative pathways, and networks. Pathway analysis through GO molecular analysis suggested that many compounds in ABR demonstrate therapeutic activity and exert similar pharmacological functions. Results of our study demonstrated the depiction of a multiple chemical-target mechanism pathway, where compounds like quercetin and rutin demonstrate similar mechanistic actions via inhibiting and activating similar pathways. The important ingredients of ABR, named as Baicalien, Copistine, Epiberberine, Kaempferol, and Palmatine, have good affinity with interleukin-6, suggesting great potential of ABR in treating bone trauma. These results provided some insight into understanding the molecular action of ABR, as well as successfully treating bone trauma and injury. This article establishes the potential therapeutic activity of ABR. Pharmacological experiments are additionally required to further prove the treatment of bone trauma by using ABR.

## Figures and Tables

**Table 1:** Node degree of ABR targets acquired via STITCH.

Target	Degree	Target	Degree
ESR1	15	PTGS2	17
MMP2	17	JUN	20
CCL2	10	ALOX5	4
MAPK14	17	FOS	18
ZFP36	5	MAPK8	17
CMA1	4	MAPK9	11
IL6	21	BCAR1	6
MMP3	14	TIMP2	7
PLA2G1B	3	IL6R	2
MMP1	16	ATF2	10
CYP1A2	3	PTPN1	8
CTSB	7	NCOA3	7
SRC	21	MAPK10	11

**Table 2:** Action view of functional targets of ABR attained via STITCH.

Targets	Activation	Inhibition	Binding	Catalysis	Post-trans. Mod.	Reaction	Expression	Score
FOS	•	•	•	•	•	•	•	0.999
MAPK8	•		•	•	•		•	0.999
MAPK9	•		•	•	•		•	0.999
BCAR1	•		•	•	•	•	•	0.999
TIMP2	•	•	•	•		•		0.999
IL6R	•		•					0.999
ATF2	•	•	•	•	•	•	•	0.999
PTPN1	•	•	•	•	•	•	•	0.999
NCOA3	•		•					0.999
MAPK10	•		•	•	•		•	0.999

**Table 3:** Functional enrichments in the PPIN.

Biological process (GO)			
Pathway ID	Pathway description	Count in gene set	False discovery rate
GO:0071310	Cellular response to organic substance	19	1.18*e*-11
GO:0031349	Positive regulation of defense response	12	3*e*-11
GO:0031347	Regulation of defense response	14	4.31*e*-11
GO:0050778	Positive regulation of immune response	13	4.31*e*-11
GO:0010243	Response to organonitrogen compound	14	8.28*e*-11
Molecular function (GO)			
Pathway ID	Pathway description	Count in gene set	False discovery rate
GO:0004705	JUN kinase activity	3	1.87*e*-06
GO:0004707	MAP kinase activity	4	1.87*e*-06
GO:0005515	Protein binding	20	1.87*e*-06
GO:0019899	Enzyme binding	12	9.59*e*-06
GO:0008134	Transcription factor binding	7	0.000272
Cellular component (GO)			
Pathway ID	Pathway description	Count in gene set	False discovery rate
GO:0005615	Extracellular space	10	0.00162
GO:0005896	Interleukin-6 receptor complex	2	0.00381
GO:0005829	Cytosol	12	0.0337
GO:0005901	Caveola	3	0.0337
GO:0044421	Extracellular region part	13	0.0337
KEGG pathways			
Pathway ID	Pathway description	Count in gene set	False discovery rate
04668	TNF signaling pathway	10	1.28*e*-14
05142	Chagas disease (American trypanosomiasis)	7	3.52*e*-09
04917	Prolactin signaling pathway	6	1.85*e*-08
05133	Pertussis	6	1.85*e*-08
05161	Hepatitis B	7	1.95*e*-08

**Table 4:** GO terms and associated genes obtained through ClueGO.

GO ID	GO term	Term *p* value (¤)	Group *p* value (¤)	Associated genes found
GO:0038128	ERBB2 signaling pathway	8.32*E*-05 (8.32*E*-04)	8.32*E*-05 (1.66*E*-04)	[KRAS, SHC1, SRC]
GO:0051353	Positive regulation of oxidoreductase activity	1.73*E*-04 (1.21*E*-03)	1.73*E*-04 (1.73*E*-04)	[CCS, ESR1, KRAS]
GO:0007254	JNK cascade	3.78*E*-10 (1.32*E*-08)	4.05*E*-14 (2.03*E*-13)	[MAP2K4, MAP2K7, MAP3K11, MAPK10, MAPK8, MAPK9, PTPN1, RB1CC1, TAOK3]
GO:0044236	Multicellular organism metabolic process	4.85*E*-07 (1.31*E*-05)	3.90*E*-08 (1.17*E*-07)	[CCL2, CTSB, MMP1, MMP2, MMP3, PLA2G1B]
GO:0031295	T cell costimulation	1.50*E*-06 (3.45*E*-05)	1.77*E*-08 (7.08*E*-08)	[CSK, DPP4, FYN, LCK, SRC]
GO:0043406	Positive regulation of MAP kinase activity	1.58*E*-12 (5.68*E*-11)	2.05*E*-18 (1.23*E*-17)	[CSK, KRAS, MAP2K4, MAP2K7, MAP3K11, MAPK10, PLA2G1B, PTPN1, SHC1, SRC, TAOK3]

Corrected with Bonferroni step down.

**Table 5:** Docking scores of various compounds with interleukin-6.

Ingredients	Docking score (KCAL/mol)	KI (mM)	Ref rmsd (A)	No. of H-bonds	H-bonds	Polar bonds	Docking outcome
Aregenal	-3.22	4.39	23.822	4	ARG179, ARG182, MET67, SER176	LYS66, GLU172	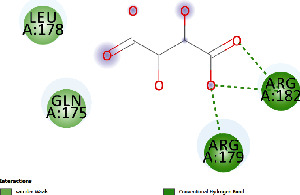
Astragalin	-5.21	152.88	29.147	4	ASN61, GLU93, LYS86, LYS66	PRO65, LEU64	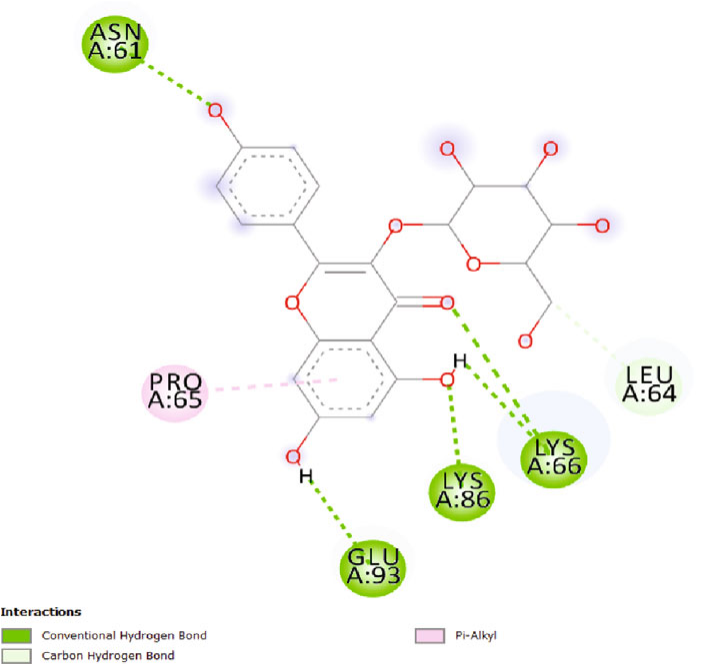
Baicalien	-6.86	9.42	29.610	6	ASN144, PRO139, GLU95, LYS120, ARG179, ARG182	LEU148, VAL45, ALA144, GLU99	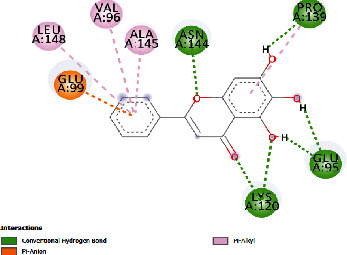
Copistine	-6.74	11.44	30.583	6	ASN63, TYR97, ASP40, LYS150, ARG179, ARG182	LEU147, THR143	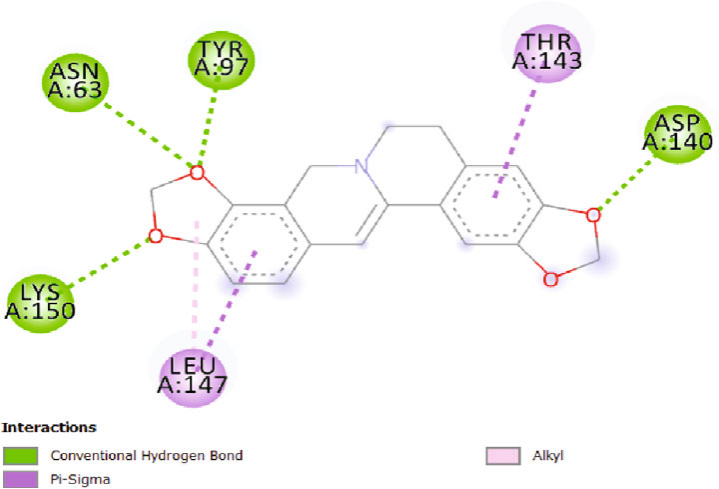
Epiberberine	-6.47	18.24	22.627	2	ARG179, ARG182	LYS66, MET67, SER176, PHE74, GLU 172	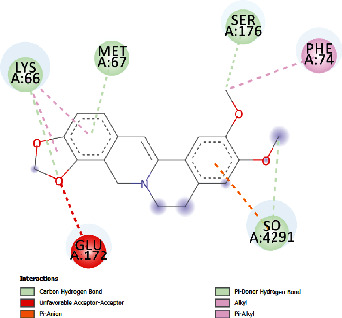
Hyperin	-4.59	432.10	30.431	4	GLU99, ARG179, ARG182, PRO139	LYS120, PRO141	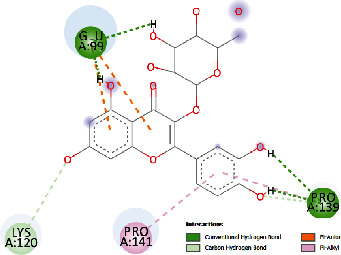
Kaempferol	-6.78	10.69	21.78	6	ARG179, ARG182, SER169, MET67, LYS66, SER176	PHE74	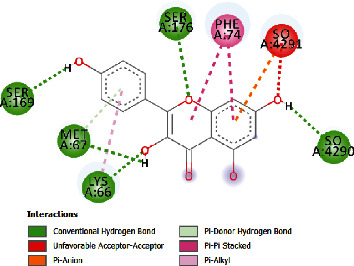
Myristic acid	-4.86	272.61	12.876	2	ARG179, ARG182	LEU178, ARG30, LEU33	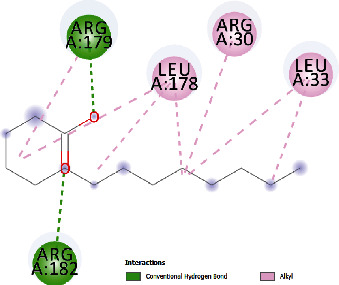
Palmatine	-6.60	14.49	31.156	3	ASN61, LYS66, LYS86	GLU93, ASN63	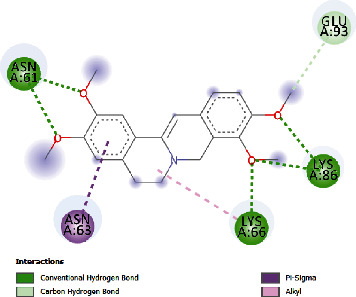
Rutin	-5.15	166.83	27.461	5	GLU99, GLN116, ASN144, LYS120, PRO139	GLU95, LEU92, THR138, PRO144	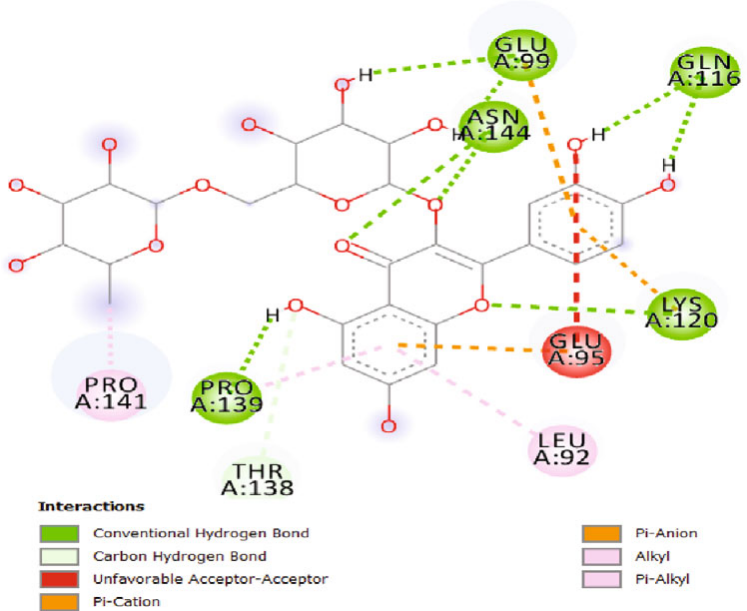

**Table 6:** Active chemical components of ABR with therapeutic effect against bone trauma.

MOL ID	Molecule name	MOL ID	Molecule name
MOL001458	Coptisine	MOL000422	Kaempferol
MOL001454	Berberine	MOL000430	Betaine
MOL001393	Myristic acid	MOL004368	Hyperin
MOL000173	Wogonin	MOL004686	Nonenone
MOL002714	Baicalein	MOL000561	Astragalin
MOL002776	Baicalin	MOL006731	Areginal
MOL002347	(R)-Allantoin	MOL000785	Palmatine
MOL000303	Caprylic acid	MOL000869	Henicosane
MOL000415	Rutin	MOL000098	Quercetin

## Data Availability

All data have been included in article.

## Supplementary Materials

Supplementary Materials Supplementary Table 1: the phytochemical ingredients obtained through TCMSP search; Supplementary Table 2: active ingredients retrieved from UniProt.
