# Supplementary material for: Network Pharmacological Study of Achyranthis bidentatae Radix Effect on Bone Trauma
Source: Biomed Res Int. 2021 Mar 6;2021:5692039. doi: 10.1155/2021/5692039 (PMC7959927; doi:10.1155/2021/5692039)
Supplement: Supplementary Materials — Supplementary Table 1: the phytochemical ingredients obtained through TCMSP search; Supplementary Table 2: active ingredients retrieved from UniProt. [file 5692039.f1.docx]

Supplementary Table 1. Phytochemical ingredients obtained through TCMSP search

| No. | MOL001006 | [poriferasta-7,22E-dien-3beta-ol](https://tcmspw.com/molecule.php?qn=1006) | 89 | MOL012533 | [quercetin-3-O-rutinoside](https://tcmspw.com/molecule.php?qn=12533) |
| --- | --- | --- | --- | --- | --- |
| 1 | MOL011389 | [chikusetsusaponin Ⅳa](https://tcmspw.com/molecule.php?qn=11389) | 90 | MOL012534 | [rhapontisterone B](https://tcmspw.com/molecule.php?qn=12534) |
| 2 | MOL011390 | [3-epioleanolic acid](https://tcmspw.com/molecule.php?qn=11390) | 91 | MOL012535 | [rubrosterone](https://tcmspw.com/molecule.php?qn=12535) |
| 3 | MOL011404 | [ginsenoside-Rg2_qt](https://tcmspw.com/molecule.php?qn=11404) | 92 | MOL012536 | [Rubschisantherin](https://tcmspw.com/molecule.php?qn=12536) |
| 4 | MOL011944 | [IPZ](https://tcmspw.com/molecule.php?qn=11944) | 93 | MOL012537 | [Spinoside A](https://tcmspw.com/molecule.php?qn=12537) |
| 56 | MOL012277 | [3-O-(β-D-glucopyranosiduronic acid) oleanolic acid](https://tcmspw.com/molecule.php?qn=12277) | 94 | MOL012538 | [stachysterone A](https://tcmspw.com/molecule.php?qn=12538) |
| 7 | MOL012451 | [(20r,22r)-2beta,3beta,20,22,26-pentahydroxy-cholestan-7,12-dien-6-one](https://tcmspw.com/molecule.php?qn=12451) | 95 | MOL012539 | [stachysterone D](https://tcmspw.com/molecule.php?qn=12539) |
| 8 | MOL012452 | [(20R,22R)-2β,3β,20,22,26-pentahydroxy-cholestan-7,12-dien-6-one](https://tcmspw.com/molecule.php?qn=12452) | 96 | MOL012540 | [zingibroside r1](https://tcmspw.com/molecule.php?qn=12540) |
| 9 | MOL012453 | [18-(β-D-Oxy glucose)-28-Oxo-12-oleanolic acid-3β-3-O-(β-D-glucose)-β-D-glucuronic acid methylester](https://tcmspw.com/molecule.php?qn=12453) | 97 | MOL012541 | [zingibroside r1_qt](https://tcmspw.com/molecule.php?qn=12541) |
| 10 | MOL012454 | [18-(β-D-Oxy glucose)-28-Oxo-12-oleanolic acid-3β-3-O-(β-D-glucose)-β-D-glucuronic acid methylester_qt](https://tcmspw.com/molecule.php?qn=12454) | 98 | MOL012542 | [β-ecdysterone](https://tcmspw.com/molecule.php?qn=12542) |
| 11 | MOL012455 | [oct-1-en-2-ol](https://tcmspw.com/molecule.php?qn=12455) | 99 | MOL001285 | [octanol](https://tcmspw.com/molecule.php?qn=1285) |
| 12 | MOL012456 | [Inokosterone](https://tcmspw.com/molecule.php?qn=12456) | 100 | MOL001314 | [Azelex](https://tcmspw.com/molecule.php?qn=1314) |
| 13 | MOL012457 | [28-deglucosyl achyranthoside D methyl ester](https://tcmspw.com/molecule.php?qn=12457) | 101 | MOL001393 | [myristic acid](https://tcmspw.com/molecule.php?qn=1393) |
| 14 | MOL012458 | [achyranthoside D trimethyl ester_qt](https://tcmspw.com/molecule.php?qn=12458) | 102 | MOL001394 | [Oktadekan](https://tcmspw.com/molecule.php?qn=1394) |
| 15 | MOL012459 | [28-deglucosyl-chikusetsusaponin,iva](https://tcmspw.com/molecule.php?qn=12459) | 103 | MOL001399 | [TWT](https://tcmspw.com/molecule.php?qn=1399) |
| 16 | MOL012460 | [28-deglucosyl-chikusetsusaponin,iva_qt](https://tcmspw.com/molecule.php?qn=12460) | 104 | MOL001454 | [berberine](https://tcmspw.com/molecule.php?qn=1454) |
| 17 | MOL012461 | [28-norolean-17-en-3-ol](https://tcmspw.com/molecule.php?qn=12461) | 105 | MOL001458 | [coptisine](https://tcmspw.com/molecule.php?qn=1458) |
| 18 | MOL012462 | [2-Octenal, 2-butyl-](https://tcmspw.com/molecule.php?qn=12462) | 106 | MOL001619 | [UPL](https://tcmspw.com/molecule.php?qn=1619) |
| 19 | MOL012463 | [2β,3β,20α,22α,25-pentahydroxy cholesta-8,14-dien-6-one](https://tcmspw.com/molecule.php?qn=12463) | 107 | MOL001655 | [oleanolic acid-3-O-β-D-glucuronopyranoside_qt](https://tcmspw.com/molecule.php?qn=1655) |
| 20 | MOL012464 | [3-O-(β-D-glucose)-oleanolic acid-28-O-(β-D-glucose)](https://tcmspw.com/molecule.php?qn=12464) | 108 | MOL000172 | [Furol](https://tcmspw.com/molecule.php?qn=172) |
| 21 | MOL012465 | [(3S,4aR,6aR,6bS,8aS,12aS,14aR,14bR)-4,4,6a,6b,11,11,14b-heptamethyl-1,2,3,4a,5,6,7,8,9,10,12,12a,14,14a-tetradecahydropicene-3,8a-diol](https://tcmspw.com/molecule.php?qn=12465) | 109 | MOL002245 | [Chrysophanol-8-O-beta-D-(6'-O-galloyl)-glucopyranoside](https://tcmspw.com/molecule.php?qn=2245) |
| 22 | MOL012466 | [3-O-(β-D-glucuronic acid)-oleanolic acid-28-O-(β-D-glucose)](https://tcmspw.com/molecule.php?qn=12466) | 110 | MOL000173 | [wogonin](https://tcmspw.com/molecule.php?qn=173) |
| 23 | MOL012467 | [3-O-β-D-glucopyranosyl-α-spinalsterol](https://tcmspw.com/molecule.php?qn=12467) | 111 | MOL001747 | [Tetracosane](https://tcmspw.com/molecule.php?qn=1747) |
| 24 | MOL012468 | [3-O-β-D-glucuronopyranoside-6-O-butyl ester](https://tcmspw.com/molecule.php?qn=12468) | 112 | MOL001836 | [n-butyl-β-D-fructopyronoside](https://tcmspw.com/molecule.php?qn=1836) |
| 25 | MOL012469 | [3-O-β-D-glucuronopyranoside-6-O-methyl ester](https://tcmspw.com/molecule.php?qn=12469) | 113 | MOL002046 | [hexanoic acid](https://tcmspw.com/molecule.php?qn=2046) |
| 26 | MOL012470 | [6-Dodecanone](https://tcmspw.com/molecule.php?qn=12470) | 114 | MOL001729 | [Crysophanol](https://tcmspw.com/molecule.php?qn=1729) |
| 27 | MOL012471 | [achybidensaponin,i](https://tcmspw.com/molecule.php?qn=12471) | 115 | MOL002347 | [(R)-Allantoin](https://tcmspw.com/molecule.php?qn=2347) |
| 28 | MOL012472 | [achybidensaponin,i_qt](https://tcmspw.com/molecule.php?qn=12472) | 116 | MOL002379 | [PTL](https://tcmspw.com/molecule.php?qn=2379) |
| 29 | MOL012473 | [achybidensaponin,ii](https://tcmspw.com/molecule.php?qn=12473) | 117 | MOL000263 | [oleanolic acid](https://tcmspw.com/molecule.php?qn=263) |
| 30 | MOL012474 | [achybidensaponin,ii_qt](https://tcmspw.com/molecule.php?qn=12474) | 118 | MOL002643 | [delta 7-stigmastenol](https://tcmspw.com/molecule.php?qn=2643) |
| 31 | MOL012475 | [achyranthesterone A](https://tcmspw.com/molecule.php?qn=12475) | 119 | MOL002714 | [baicalein](https://tcmspw.com/molecule.php?qn=2714) |
| 32 | MOL012476 | [achyranthoside Ⅱ](https://tcmspw.com/molecule.php?qn=12476) | 120 | MOL002776 | [Baicalin](https://tcmspw.com/molecule.php?qn=2776) |
| 33 | MOL012477 | [achyranthoside Ⅱ_qt](https://tcmspw.com/molecule.php?qn=12477) | 121 | MOL002897 | [epiberberine](https://tcmspw.com/molecule.php?qn=2897) |
| 34 | MOL012478 | [achyranthoside Ⅲ](https://tcmspw.com/molecule.php?qn=12478) | 122 | MOL000303 | [caprylic acid](https://tcmspw.com/molecule.php?qn=303) |
| 35 | MOL012479 | [achyranthoside Ⅳ](https://tcmspw.com/molecule.php?qn=12479) | 123 | MOL003040 | [Amylol](https://tcmspw.com/molecule.php?qn=3040) |
| 36 | MOL012480 | [achyranthoside A](https://tcmspw.com/molecule.php?qn=12480) | 124 | MOL003091 | [pent-3-en-2-one](https://tcmspw.com/molecule.php?qn=3091) |
| 37 | MOL012481 | [achyranthoside A_qt](https://tcmspw.com/molecule.php?qn=12481) | 125 | MOL000346 | [succinic acid](https://tcmspw.com/molecule.php?qn=346) |
| 38 | MOL012482 | [achyranthoside A trimethyl ester](https://tcmspw.com/molecule.php?qn=12482) | 126 | MOL003507 | [Heptanol](https://tcmspw.com/molecule.php?qn=3507) |
| 39 | MOL012483 | [achyranthoside A trimethyl ester_qt](https://tcmspw.com/molecule.php?qn=12483) | 127 | MOL000357 | [Sitogluside](https://tcmspw.com/molecule.php?qn=357) |
| 40 | MOL012484 | [achyranthoside c](https://tcmspw.com/molecule.php?qn=12484) | 128 | MOL000358 | [beta-sitosterol](https://tcmspw.com/molecule.php?qn=358) |
| 41 | MOL012485 | [achyranthoside c_qt](https://tcmspw.com/molecule.php?qn=12485) | 129 | MOL003702 | [geniposide_qt](https://tcmspw.com/molecule.php?qn=3702) |
| 42 | MOL012486 | [achyranthoside C butyl dimethyl ester](https://tcmspw.com/molecule.php?qn=12486) | 130 | MOL003778 | [pjs-1_qt](https://tcmspw.com/molecule.php?qn=3778) |
| 43 | MOL012487 | [achyranthoside C butyl dimethyl ester_qt](https://tcmspw.com/molecule.php?qn=12487) | 131 | MOL000383 | [D-Galacturonic acid, homopolymer](https://tcmspw.com/molecule.php?qn=383) |
| 44 | MOL012488 | [achyranthoside C dimethyl ester](https://tcmspw.com/molecule.php?qn=12488) | 132 | MOL003847 | [Inophyllum E](https://tcmspw.com/molecule.php?qn=3847) |
| 45 | MOL012489 | [achyranthoside C dimethyl ester_qt](https://tcmspw.com/molecule.php?qn=12489) | 133 | MOL000415 | [rutin](https://tcmspw.com/molecule.php?qn=415) |
| 46 | MOL012490 | [achyranthoside C trimethyl ester](https://tcmspw.com/molecule.php?qn=12490) | 134 | MOL000422 | [kaempferol](https://tcmspw.com/molecule.php?qn=422) |
| 47 | MOL012491 | [achyranthoside D](https://tcmspw.com/molecule.php?qn=12491) | 135 | MOL000430 | [betaine](https://tcmspw.com/molecule.php?qn=430) |
| 48 | MOL012492 | [achyranthoside D trimethyl ester](https://tcmspw.com/molecule.php?qn=12492) | 136 | MOL004355 | [Spinasterol](https://tcmspw.com/molecule.php?qn=4355) |
| 49 | MOL012493 | [achyranthoside E](https://tcmspw.com/molecule.php?qn=12493) | 137 | MOL000449 | [Stigmasterol](https://tcmspw.com/molecule.php?qn=449) |
| 50 | MOL012494 | [achyranthoside E_qt](https://tcmspw.com/molecule.php?qn=12494) | 138 | MOL012511 | [Chikusetsusaponin V methyl ester](https://tcmspw.com/molecule.php?qn=12511) |
| 51 | MOL012495 | [achyranthoside E butyl dimethyl ester](https://tcmspw.com/molecule.php?qn=12495) | 139 | MOL004664 | [heptanoic acid](https://tcmspw.com/molecule.php?qn=4664) |
| 52 | MOL012496 | [achyranthoside E butyl dimethyl ester_qt](https://tcmspw.com/molecule.php?qn=12496) | 140 | MOL004368 | [Hyperin](https://tcmspw.com/molecule.php?qn=4368) |
| 53 | MOL012497 | [achyranthoside E dimethyl ester](https://tcmspw.com/molecule.php?qn=12497) | 141 | MOL004686 | [Nonenone](https://tcmspw.com/molecule.php?qn=4686) |
| 54 | MOL012498 | [achyranthoside E dimethyl ester_qt](https://tcmspw.com/molecule.php?qn=12498) | 142 | MOL005155 | [ginsenoside Ro_qt](https://tcmspw.com/molecule.php?qn=5155) |
| 55 | MOL012499 | [achyranthoside E trimethyl ester](https://tcmspw.com/molecule.php?qn=12499) | 143 | MOL005325 | [ginsenoside Ro](https://tcmspw.com/molecule.php?qn=5325) |
| 56 | MOL012500 | [achyranthoside E trimethyl ester_qt](https://tcmspw.com/molecule.php?qn=12500) | 144 | MOL000561 | [Astragalin](https://tcmspw.com/molecule.php?qn=561) |
| 57 | MOL012501 | [achyranthoside I](https://tcmspw.com/molecule.php?qn=12501) | 145 | MOL000057 | [DIBP](https://tcmspw.com/molecule.php?qn=57) |
| 58 | MOL012502 | [bidentatoside,i](https://tcmspw.com/molecule.php?qn=12502) | 146 | MOL000666 | [hexanal](https://tcmspw.com/molecule.php?qn=666) |
| 59 | MOL012503 | [bidentatoside,i_qt](https://tcmspw.com/molecule.php?qn=12503) | 147 | MOL000667 | [1-hexanol](https://tcmspw.com/molecule.php?qn=667) |
| 60 | MOL012504 | [bidentatoside,ii](https://tcmspw.com/molecule.php?qn=12504) | 148 | MOL006731 | [Areginal](https://tcmspw.com/molecule.php?qn=6731) |
| 61 | MOL012505 | [bidentatoside,ii_qt](https://tcmspw.com/molecule.php?qn=12505) | 149 | MOL000676 | [DBP](https://tcmspw.com/molecule.php?qn=676) |
| 62 | MOL012506 | [chikusetsusaponin Ⅳ](https://tcmspw.com/molecule.php?qn=12506) | 150 | MOL000069 | [palmitic acid](https://tcmspw.com/molecule.php?qn=69) |
| 63 | MOL012507 | [chikusetsusaponin I](https://tcmspw.com/molecule.php?qn=12507) | 151 | MOL000703 | [2-heptanone](https://tcmspw.com/molecule.php?qn=703) |
| 64 | MOL012508 | [chikusetsusaponin IVA butyl ester](https://tcmspw.com/molecule.php?qn=12508) | 152 | MOL000708 | [WLN: VHR](https://tcmspw.com/molecule.php?qn=708) |
| 65 | MOL012509 | [Chikusetsusaponin IVa methyl ester](https://tcmspw.com/molecule.php?qn=12509) | 153 | MOL000713 | [(E)-oct-3-en-2-one](https://tcmspw.com/molecule.php?qn=713) |
| 66 | MOL012510 | [Chikusetsusaponin V butyl ester](https://tcmspw.com/molecule.php?qn=12510) | 154 | MOL000748 | [HMF](https://tcmspw.com/molecule.php?qn=748) |
| 67 | MOL004652 | [(2R,3R,4S,5S,6R)-2-[[(3S,5S,9R,10S,13R,14R,17R)-17-[(E,2R,5S)-5-ethyl-6-methylhept-3-en-2-yl]-10,13-dimethyl-2,3,4,5,6,9,11,12,14,15,16,17-dodecahydro-1H-cyclopenta[a]phenanthren-3-yl]oxy]-6-(hydroxymethyl)oxane-3,4,5-triol](https://tcmspw.com/molecule.php?qn=4652) | 155 | MOL007485 | [(2S,3S,4S,5R,6R)-6-[[(3S,4aR,6aR,6bS,8aS,12aS,14aR,14bR)-4,4,6a,6b,11,11,14b-heptamethyl-8a-[oxo-[[(2S,3R,4S,5S,6R)-3,4,5-trihydroxy-6-(hydroxymethyl)-2-tetrahydropyranyl]oxy]methyl]-1,2,3,4a,5,6,7,8,9,10,12,12a,14,14a-tetradecahydropicen-3-yl]oxy]-3,4-di](https://tcmspw.com/molecule.php?qn=7485) |
| 68 | MOL012512 | [deglucose chikusetsusaponin Iva](https://tcmspw.com/molecule.php?qn=12512) | 156 | MOL000775 | [EEE](https://tcmspw.com/molecule.php?qn=775) |
| 69 | MOL012513 | [deglucose chikusetsusaponin Iva_qt](https://tcmspw.com/molecule.php?qn=12513) | 157 | MOL000776 | [OXA](https://tcmspw.com/molecule.php?qn=776) |
| 70 | MOL012514 | [Ecdysterone-3-O-beta-D-glucopyranoside](https://tcmspw.com/molecule.php?qn=12514) | 158 | MOL000785 | [palmatine](https://tcmspw.com/molecule.php?qn=785) |
| 71 | MOL012515 | [Ecdysterone-3-O-beta-D-glucopyranoside_qt](https://tcmspw.com/molecule.php?qn=12515) | 159 | MOL007891 | [2,6-Dimethylpiazine](https://tcmspw.com/molecule.php?qn=7891) |
| 72 | MOL012516 | [geniposide](https://tcmspw.com/molecule.php?qn=12516) | 160 | MOL008279 | [Amyl ketone](https://tcmspw.com/molecule.php?qn=8279) |
| 73 | MOL012517 | [hederagenin-28-O-β-D-glucopyranosyl ester](https://tcmspw.com/molecule.php?qn=12517) | 161 | MOL000084 | [beta-daucosterol](https://tcmspw.com/molecule.php?qn=84) |
| 74 | MOL012518 | [hederagenin-28-O-β-D-glucopyranosyl ester_qt](https://tcmspw.com/molecule.php?qn=12518) | 162 | MOL000085 | [beta-daucosterol_qt](https://tcmspw.com/molecule.php?qn=85) |
| 75 | MOL012519 | [Monoglucuronide F](https://tcmspw.com/molecule.php?qn=12519) | 163 | MOL000860 | [stearic acid](https://tcmspw.com/molecule.php?qn=860) |
| 76 | MOL012520 | [niuxixinsterone A](https://tcmspw.com/molecule.php?qn=12520) | 164 | MOL000863 | [Dekan](https://tcmspw.com/molecule.php?qn=863) |
| 77 | MOL012521 | [niuxixinsterone B](https://tcmspw.com/molecule.php?qn=12521) | 165 | MOL000865 | [hexadecane](https://tcmspw.com/molecule.php?qn=865) |
| 78 | MOL012522 | [niuxixinsterone C](https://tcmspw.com/molecule.php?qn=12522) | 166 | MOL008653 | [Acetylfuran](https://tcmspw.com/molecule.php?qn=8653) |
| 79 | MOL012523 | [N-trans-feruloyl-3-methoxytyramine-4'-O-β-D-glucopyranoside](https://tcmspw.com/molecule.php?qn=12523) | 167 | MOL012527 | [oleanolic acid 3-O-[β-D-glucuronopyranoside-6-O-ethyl ester]-28-O-β-D-glucopyranoside](https://tcmspw.com/molecule.php?qn=12527) |
| 80 | MOL012524 | [Hmp-hmpep](https://tcmspw.com/molecule.php?qn=12524) | 168 | MOL012528 | [oleanolic acid 3-O-[β-D-glucuronopyranoside-6-O-methyl ester]-28-O-β-D-glucopyranoside](https://tcmspw.com/molecule.php?qn=12528) |
| 81 | MOL012525 | [N-trans-feruloyl-3-methoxytyramine-4-O-β-D-glucopyranoside](https://tcmspw.com/molecule.php?qn=12525) | 169 | MOL012529 | [oleanolic acid-3-O-β-D-(6'-butyl)-glucuronopyranoside](https://tcmspw.com/molecule.php?qn=12529) |
| 82 | MOL012526 | [oleanolic acid 3-O-[β-D-glucuronopyranoside-6-O-butyl ester]-28-O-β-D-glucopyranoside](https://tcmspw.com/molecule.php?qn=12526) | 170 | MOL012530 | [oleanolic acid-3-O-β-D-glucuronopyranoside](https://tcmspw.com/molecule.php?qn=12530) |
| 83 | MOL000867 | [Heptadekan](https://tcmspw.com/molecule.php?qn=867) | 171 | MOL000876 | [(6R,10R)-6,10,14-trimethylpentadecan-2-one](https://tcmspw.com/molecule.php?qn=876) |
| 84 | MOL008671 | [PRZ](https://tcmspw.com/molecule.php?qn=8671) | 172 | MOL000879 | [methyl palmitate](https://tcmspw.com/molecule.php?qn=879) |
| 85 | MOL008680 | [acetaldehyde](https://tcmspw.com/molecule.php?qn=8680) | 173 | MOL000880 | [Tricosane](https://tcmspw.com/molecule.php?qn=880) |
| 86 | MOL000869 | [Henicosane](https://tcmspw.com/molecule.php?qn=869) | 174 | MOL000885 | [Dodekan](https://tcmspw.com/molecule.php?qn=885) |
| 87 | MOL012531 | [pjs-1](https://tcmspw.com/molecule.php?qn=12531) | 175 | MOL000886 | [tetradecane](https://tcmspw.com/molecule.php?qn=886) |
| 88 | MOL012532 | [Polypodine B](https://tcmspw.com/molecule.php?qn=12532) | 176 | MOL000098 | [quercetin](https://tcmspw.com/molecule.php?qn=98) |

Supplementary Table 2. Active ingredients retrieved from UniProt

| No. | Targets | No. | Targets |
| --- | --- | --- | --- |
| 1 | [Interleukin-6](https://tcmspw.com/target.php?qt=351) | 12 | [Collagen alpha-1(I) chain](https://tcmspw.com/target.php?qt=731) |
| 2 | [Interstitial collagenase](https://tcmspw.com/target.php?qt=353) | 13 | [Prostaglandin G/H synthase 2](https://tcmspw.com/target.php?qt=94) |
| 3 | [Cathepsin B](https://tcmspw.com/target.php?qt=361) | 14 | [Thioredoxin reductase, cytoplasmic](https://tcmspw.com/target.php?qt=538) |
| 4 | [Estrogen receptor](https://tcmspw.com/target.php?qt=46) | 15 | [Cytochrome P450 1A2](https://tcmspw.com/target.php?qt=724) |
| 5 | [Mitogen-activated protein kinase 14](https://tcmspw.com/target.php?qt=402) | 16 | [Dihydroorotate dehydrogenase, mitochondrial](https://tcmspw.com/target.php?qt=756) |
| 6 | [Chymase](https://tcmspw.com/target.php?qt=412) | 17 | [Elastase 1](https://tcmspw.com/target.php?qt=698) |
| 7 | [Transcription factor AP-1](https://tcmspw.com/target.php?qt=414) | 18 | [Phospholipase A2](https://tcmspw.com/target.php?qt=708) |
| 8 | [C-C motif chemokine 2](https://tcmspw.com/target.php?qt=417) | 19 | [72 kDa type IV collagenase](https://tcmspw.com/target.php?qt=238) |
| 9 | [Stromelysin-1](https://tcmspw.com/target.php?qt=441) | 20 | [Tumor necrosis factor](https://tcmspw.com/target.php?qt=265) |
| 10 | [CGMP-specific 3',5'-cyclic phosphodiesterase](https://tcmspw.com/target.php?qt=65) | 21 | [Proto-oncogene tyrosine-protein kinase SRC](https://tcmspw.com/target.php?qt=319) |
| 11 | [Arachidonate 5-lipoxygenase](https://tcmspw.com/target.php?qt=88) |  |  |
